# What is a data space—Logical architecture model

**DOI:** 10.1016/j.dib.2025.111575

**Published:** 2025-04-21

**Authors:** Juha-Pekka Soininen, Gabriella Laatikainen

**Affiliations:** a VTT Technical Research Centre of Finland, Kaitoväylä 1, PO. Box 1100, FI-90571 Oulu, Finland; b VTT Technical Rearch Centre of Finland, Koivurannantie 1, PO Box 1603, FI-40101 Jyväskylä, Finland

**Keywords:** Data sharing, Logical model, Use case model, System architecture

## Abstract

The paper presents a use case model and a logical architecture model of a data space system. The models view the data space system from the user and operator perspectives and describe the needed functionalities and their connection on an abstract level. The core features in our data space model are collaboration networks and contract-based data sharing. The models are meant as a simple and explainable first introduction to what a data space system is, and they provide an implementation technology-independent basis for creating data space implementation specifications. The model is validated by comparing it with existing data space reference models from IDSA, GAIA-X, and the Data Space Support Centre. The modelled core features map with the technical specifications of the system. Our study enables data space system developers to think out of the box and create new innovative solutions.

## 1 Introduction

Data sharing has been the primary use case of the Internet since its start in the 1970s. File transfer protocol and email were among the very first widely spread applications that allowed the transfer of digital data, information, and knowledge between computers and from one user to another [1]. During these early years, the data was converted to information and knowledge and enabling interoperability between users was accomplished using common syntaxes, data formats, and standards. Semantic information models added a link to the meaning of the data, i.e., ontology models, to the value of the data and made it possible for machines to understand the meaning of data [2]. The hypertext and world-wide-web added the address of the location of the data into data elements and enabled the seamless data sharing using Linked Data and semantic web concepts [3].

The value of data sharing has been demonstrated, especially with the Open Data concept, and opening the data for others has enabled a considerable boost in innovations and new services in almost every business domain [4]. FAIR (findable, accessible, interoperable, reusable) principles [5] have been developed to support the creation of open data. However, there is information that cannot be shared as open data. Personal and sensitive data, confidential data, and secret data are the most common examples that require consent from the target of the data [6]. There are also regulations, such as the General Data Protection Regulation (GDPR) [7] in the EU that define how data needs to be managed. Confidential or private data is typically data related to an organisation's business or operations and contains information critical for the organisation's competitiveness. The use of confidential data has been controlled by bilateral agreements such as non-disclosure agreements, but recently, especially in the EU, new legislations such as the Data Act [8] and Data Governance Act [9] have been introduced. With the development of artificial intelligence and large language models, the role of data as a business asset has become apparent, and data rights have become very important [10]. The data economy is expected to be a multi-billion Euro business globally [11]. Secret data typically refers to national security information or any other information under strict protection, and disclosure requires some kind of authorisation [12].

Today, data space refers to solutions that allow the sharing of confidential and sensitive data between legal entities, i.e., companies, organisations, and persons [13]. It also allows open data sharing, or combining confidential data with open data, while its main advantage and purpose relies on sharing confidential data. The data space concept was first introduced in [14] as a solution to enable data sharing in physical space. The Internet of Things (IoT) created data platforms that enabled physical places to be linked to digital systems. The SOFIA platform had a semantic information broker concept for sharing device information for intelligent local applications [15]. Later, the concept was detached from physical spaces, and the focus turned to the type of data that was shared. The idea in the Industrial Data Space Initiative renamed International Data Space Association (IDSA) in 2015, was to extend the LinkedData concept [16] so that confidential information could be shared between industrial companies by adding trust creation and security solutions. Design principles of data spaces were first defined in a position paper in the OpenDEI project with a strong focus on data sovereignty and the trustworthiness of data sharing infrastructure [17]. Since then, the data space has been developed by the International Data Space Association (IDSA) [18], and Gaia-X European Association for Data and Cloud AISBL (GAIA-X) [19]. Both associations are non-profit organisations developing data space specifications. The Data Space Support Centre (DSSC) project was created as part of the European activities to coordinate and specify the data spaces [20]. It has created the blueprint documents to guide the data space community in the creation of data spaces, especially in the context of common European data spaces [21]. EU-funded procurement project SIMPL is developing an open-source, secure middleware platform that supports data access and interoperability in European data initiatives including data spaces [22].

The amount of data is expected to increase every year by 120 zettabytes, so data is an incredible resource for new knowledge for the benefit of people, companies, and society. The business value of data is expected to increase to 1.75 trillion dollars by 2030. Based on these numbers, it is clear that data sharing and data use play a key role in the world now and in all domains in the future [11].

Data spaces result from various developments in data management and sharing domains. The term itself has a variety of definitions, from a large-scale pool of data from the EU and BDVA to a focused contract-based data-sharing method used in this paper. Various deployment scenarios have been proposed, from centralised platform concepts to decentralised autonomous organisation approaches. Multiple business use cases could use data space from small, closed, few-party collaborations to common European data space spanning domains such as mobility or manufacturing [23].

**Fig. 1:**
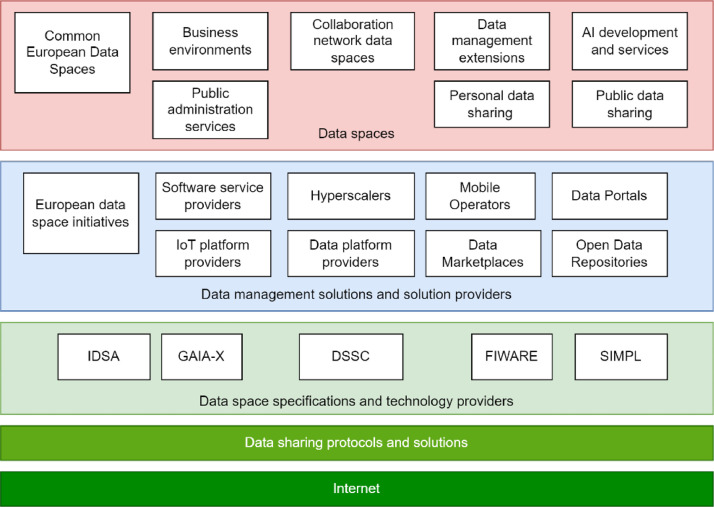
Background and evolution scenarios for data spaces based on the Internet and data sharing. The diagram shows the main categories of technologies and data management solutions and solution providers, as well as possible outcomes of the use of various approaches.

Fig. 1 presents a simplified “evolution diagram” of data spaces and provides example scenarios of potential data space usage types. It shows that data spaces can be understood in multiple ways, and different types of technology and solutions providers have different objectives and preferences when making implementations. There are actions towards convergence of approaches, as the Data Space Business Alliance [24], and ongoing standardisation activities [25], but the future will be defined by the actions that implement and provide data spaces to users.

The focus of this paper is to present a logical, implementation-independent model of the data space that, as far as we know, does not exist and could apply to all data space approaches. Data Space development has been very implementation-focused, which has led to specifications and models that use typical software system components, technologies, and standards that hide the original purpose and core functionality of data spaces. The value of a logical architecture model is that it describes the pure functionality of the data space system without any implementation dependencies. It allows one to focus on simplicity and explainability, and understand the concept of data spaces. It is a good basis for creative thinking when implementing such a system. It serves as a learning material when the data spaces are introduced. There have been some attempts to describe what data spaces and data economy are about. Oliveira et al. [26] have created a meta-model of the data economy that focuses on ecosystem players and components but lacks the definitions of functionality. Hutterer et al. [27] have developed definitions of data space and its concepts based on analysis of articles written by others. Schleimer et al. (2023) studied architecture alternatives of data space using an implementation-independent approach, but the analysis stays on a general level and does not go to actual functionalities. Sitra [28] has defined rules for the FAIR data economy that list the main principles of data sharing but do not specify actual functionalities.

The contents of the paper are as follows. In Chapter 2, we introduce the most common data-sharing approaches and the main concepts and principles of data spaces. Chapter 3 describes the logical architecture model approach. Chapter 4 presents our data space models starting from a basic use case and showing the data space environment and logical architecture models. Chapter 5 maps the logical model elements to GAIA-X, IDSA, and DSSC reference architectures and models. Chapters 6 give a discussion about how the model could be used and extended, and Chapter 7 concludes the paper.

## 2 Data Sharing

The business or use case requirements define what kind of data-sharing approach is feasible, and several parameters need to be considered. There are technical parameters that describe how the actual data can be transmitted from one company to the other, but they do not affect the logical characteristics of data-sharing approaches. Naturally, we need to have functions for transferring data, but the differences in those functions come from implementation needs. From a logical perspective, it is more interesting to consider what kind of users and data are shared, the characteristics of the collaboration network, and what kind of usage rights to data are shared, which relates to the needed contracts between the network parties.

The main types of business cases related to collaboration networks are:

• Pre-defined collaboration networks where parties know each other before the data-sharing process is started. These networks can be either static or dynamic, but the network management and trust in the parties is separated from the actual data sharing. Typical examples of such business cases are supplier networks and asset management solutions.
• Open business cases, where collaboration is set up dynamically without an existing relationship between parties. In these business cases, the discovery of data and trust between the parties must be created at the start of the data-sharing process. Typical examples are marketplaces, where products are offered to everyone or a specific category of customers.

**Fig. 2:**
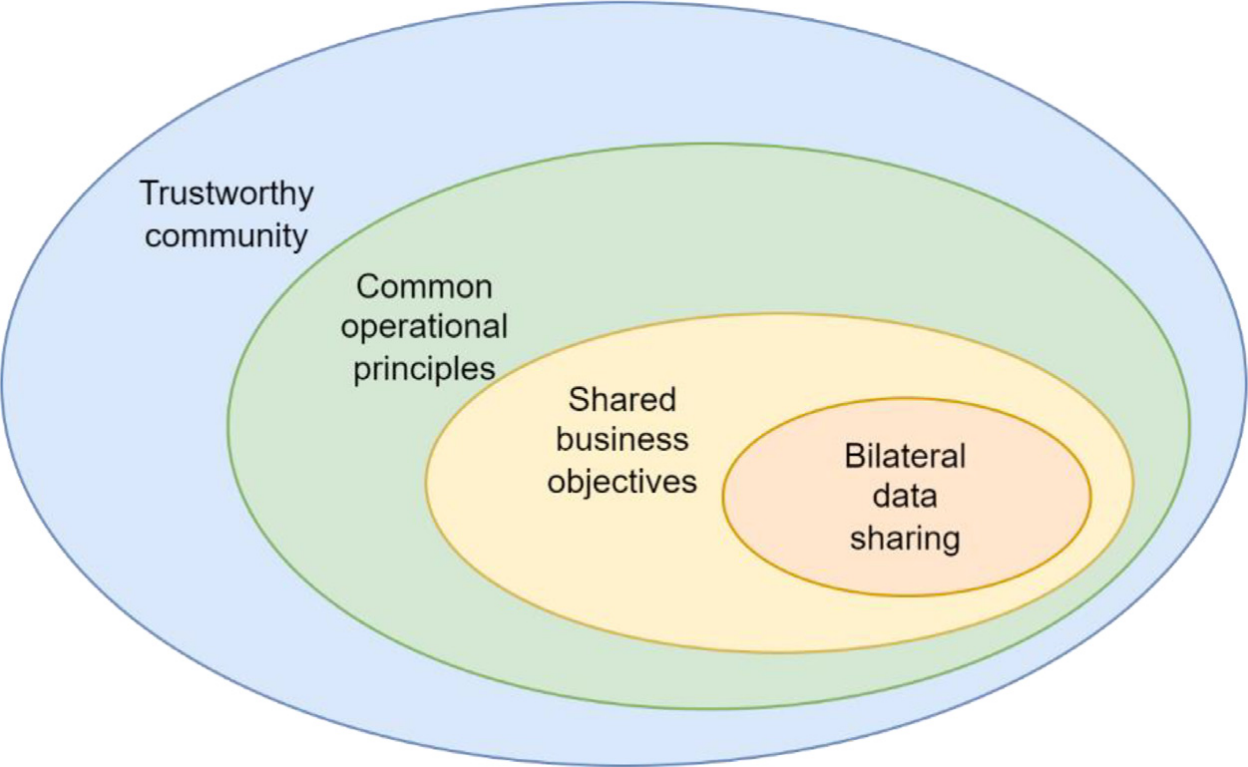
Collaboration networks in data sharing.

When thinking of collaboration networks, it is important to note the main factors affecting the creation of confidential data sharing as depicted in Fig. 2. Bilateral data sharing requires sharing business objectives, which requires common operational practices, and before such practices can be agreed upon, there must be a trustworthy community. Trustworthiness or common practices as such do not create data sharing, but there needs to be incentives that motivate companies to invest in establishing collaboration networks.

There are many ways to do data classification with varying criteria, for example, based on sensitivity, types and business context. In the case of data sharing, data can be divided, for example, into public or open, sensitive, personal, internal or confidential, and secret or restricted data [29]. The data in every category is nowadays linked with licenses that need to be accepted using contracts. In the case of public and open data, these processes are straightforward and often very simple. Sensitive and personal data sharing is governed by legislation such as GDPR [7] in Europe. In confidential business data, the transfer of data rights must be managed with a legal contract between the parties, and the monitoring of the execution of the contract must be implemented as a part of the data-sharing process [30].

### 2.1 Approaches for sharing of confidential data

**Fig. 3:**
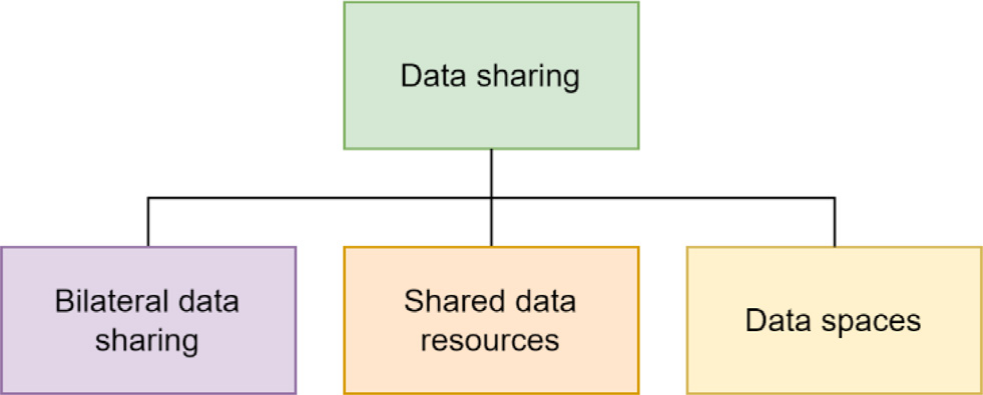
Data sharing approaches.

The main categories of data sharing approaches direct data sharing parties, i.e. bilateral data sharing, using shared data resources, and data spaces, are depicted in Fig. 3.

Bilateral data sharing happens between the data provider and the data consumer using the methods and approaches of the parties. The two basic technologies are message-based data sharing and allowing consumers to access a provider system API. Both approaches work fine in small and static collaboration networks. Message-based approaches scale better and allow more dynamism in collaboration networks. API-based networks support better, more complex data and more dynamic data needs. In the API-based approach, the challenges come from adding new members to a network and from a variety of possible APIs [31].

Shared data resources use shared data storage services from a third party. The basic mechanism is to allow the consumer to access the data storage through an API that it provides. Many solution categories have different characteristics. Typically, these solutions are provided as a service by service providers.

Shared data storages are typically cloud storages [32] where the data consumer can control access to the data. Data portals [33] extend storage with data management solutions to help data providers manage their data. Data platforms [34] provide additional data services that help, for example, to transform or analyse the data so that it fits better for the consumer or support data sharing contract management. Data lakes [35] and data meshes [36] provide unified data management to multiple data sources and storage technologies, but from a data sharing perspective, they are shared data storage controlled by a single entity. The main challenge with shared resources is the security concerns related to having data critical to a company in a resource that may be vulnerable and hosted by a cloud provider. Other challenges are related to the management of the collaboration network, which is the responsibility of the data provider, and scalability, which is limited by shared storage interfaces and possibly limited to the inside of the data platform.

The third option, data space, combines the benefits of the other two and adds the collaboration network and contract management capabilities into the technical data-sharing approach. Data spaces offer P2P data transfer capabilities like bilateral APIs, common but flexible collaboration network management and common contract management approaches that are possible with data platform services [6].

#### 2.1.1 Self-Sovereign identity (SSI) and digital wallets

One approach for confidential data sharing is related to Self-Sovereign Identity (SSI) and digital wallets that enable sharing and verifying the authenticity of identity and identity-related data. The key components of SSI include a digital wallet (i.e., an application for storing digital assets, and/or identity information; [37,38]), verifiable credentials (i.e., a tamper-evident credential that has authorship and can be cryptographically verified; [39]) and decentralised identifiers (i.e., references to information about the owners’ public keys and associated metadata; [40]). Further, many SSI implementations rely on distributed ledger technologies (a broader class of “blockchain-inspired” technology; [41]).

Successful implementation of SSI requires an ecosystem of three distinct types of actors with specific roles that form “the digital trust triangle”: the holder, issuer, and verifier [42]. The holders request credentials (i.e., the digital representation of information) from issuers and hold them in their local devices or the cloud [43]. When the verifier requests, holders can approve the request and present the credentials for verification. The issuers define the credentials, their meaning and the method for verification. Verifiers request the necessary credentials and follow their own policies to verify their authenticity [44].

#### 2.1.2 Personal online datastore (Pods)

Decentralised data stores, called Pods, are being developed primarily by the Solid community [45]. Solid is an open standard for structuring data and applications on the internet. Solid adheres to the human-centric paradigm that aims to give control of data to the data owner. Solid proposes the idea of Pods that act as secure web servers, controlled by the data owner. The data stored in Solid Pods supports the Linked Data design principles that enable the sharing of interlinked data across multiple applications. Pods allow users to decide who can access their data, what data is shared, and for what purposes.

### 2.2 Key concepts of data spaces

Data space has had many definitions in the literature [46]. We list the definitions found in the literature in Table 1.

**Table 1:** Data space definitions.

Definition	Source
“Interoperable framework, based on common governance principles, standards, practices and enabling services, enables trusted data transactions between participants.”	[47]
“any ecosystem of data models, datasets, ontologies, data sharing contracts and specialised management services (i.e., as often provided by data centres, stores, repositories, individually or within ‘data lakes’), together with soft competencies around it (i.e., governance, social interactions, business processes).”	[48]
“A dataspace system manages the large-scale heterogeneous collection of data distributed over various data sources in different formats. It addresses the structured, semi-structured, and unstructured data in a coordinated manner without presuming the semantic integration among them.”	[49]
“Dataspace is defined as a set of participants and a set of relationships among them.”	[50]
“A data space is defined as federated data management (i.e. federation of the data platforms/sources of the various business actors).”	[51]
“Data spaces are decentralised data infrastructures designed to enable data-sharing scenarios across organisational boundaries by implementing mechanisms for secure and trustworthy data sharing—such as distributed data storage and meta-data sharing. They guarantee data sovereignty by ensuring that the data provider determines control over the access and use of the shared data.”	[52]
“data spaces can be understood as intermediaries and data sharing service providers to which the EU Data Governance Act applies”	[53]

DSSC Glossary defines the Governance Framework as “the structured set of principles, processes, and practices that guide and regulate the governance, management, and operations within a data space to ensure effective and responsible leadership, control, and oversight. It defines the functionalities the data space provides and the associated data space roles, including the data space governance authority, service intermediaries such as data space operator, and participants.”

We can discuss the key concepts of data spaces based on the key definitions.

#### 2.2.1 Data sovereignty

Data sovereignty is defined as “the ability of individuals, organisations, and governments to have control over their data and exercise their rights to the data, including its collection, storage, sharing, and use by others” [47].

The definition is abstract, leaving much room for different architectures and implementations. The main vision behind the definition is the sharing of data between parties across the Internet, so that the parties that hold the rights to the data rights are respected, and this is achieved by commitment to common rules, trust, and security [27,54].

#### 2.2.2 Data products

Data sovereignty and data holder’s rights cannot be discussed without identifying the data in question. Data itself or digital data is a collection of bits that represent information or knowledge [55]. Associating the data holder’s rights to the data requires that the data is a uniquely identifiable artefact. In this paper, we borrow the concept of *data products* from data mesh architectures [56] to refer to this artefact. A data product is the object of a *data transaction*.

#### 2.2.3 Users and members of data spaces

Data spaces are based on common rules [57]. Common rules cannot be agreed upon without knowing who agrees. The data rights holder must be a known entity too. Both common rules, the enforcement of those rules and legal rights to data require that the known entities are legal entities. To be able to trust that those legal entities are who they claim to be, we must be able to verify their identities from the legal entity identity providers. A legal entity identity provider is an example of a *Trust Anchor*, which represents an ultimate source of fact [58].

The commitment to common rules must also be verifiable. How commitment is implemented is not defined but should be legally binding. The commitment implements a new identity or an attribute to an identity that is *a member of a data space*.

#### 2.2.4 Trustworthiness

Trustworthiness stems from the capability to verify the claims presented by entities involved in the collaboration, and the capability to verify the actions done by entities when executing the data transactions and using the data. In the data spaces, these entities are the companies, the legal entities involved in data transactions, and the software components that comprise the digital infrastructure. It must be possible to verify from the data space governance that the membership claims are valid, and that the memberships are given to real legal entities from the authorities of respective countries. The trustworthiness of the software components is based on the transparency of their implementations or their immutability after validation by a governance authority. Direct verification can be implemented by using open-source components. Indirect verification can be based on audits and certification processes [46].

Regarding the execution of data transaction contracts, there needs to be a means of logging the activities defined in contracts so that both parties have the same information available both during and after the transaction.

### 2.3 Data space specifications

International Data Space Association (IDSA) has created a data space specification and reference architecture 4. The reference model has a business layer, a functional layer, an information layer, a process layer, and a system layer. The system layer has a system model that consists of data space connectors, metadata broker, clearing house, app store, vocabulary hub, and identity provider, which are divided into certificate authority, dynamic attribute provisioning service, and partner information service. The data space operator, called IDSA Support Organisation (IDSA SO) accepts all these components. IDSA Support Organisation is also responsible for creating trust. Data space connectors provide users with access to data space services and create and verify identities with the help of an identity provider. Clearing house provides event logging and storage services. The App Store provides a means to add trustworthy services to connectors. Vocabulary hub provides data interoperability services. The metadata broker contains the metadata for the available data products. The data transfers go from user to user through the control of the data space connector [59].

GAIA-X association has developed a reference architecture model based on federated services, well-defined data formats and models, and distributed identity management. The objective has been to implement a solution using an all-software approach operated by service federators, which could create trustworthiness through trust by design. Still, the architecture can be implemented using service providers as well. The GAIA-X operating model is based on a set of entities that want to share data in a GAIA-X-compliant way. The trustworthiness is based on trust anchors defined by governance partners, compliance rules that can be extended with GAIA-X labels, verifiable GAIA-X self-descriptions, and the GAIA-X trust framework that validates the claims in self-descriptions. GAIA-X registry is a database that enables the governance [60].

Data Space Support Centre (DSSC) is an EU project aiming to create common high-level specifications and a library of building blocks for data space implementations. The objective is to support the data space creation process, especially the common European data spaces. DSSC model combines the approaches presented in OPENDEI, IDSA, GAIA-X, and FIWARE [61]. Its high-level specification is based on two sets of building blocks: Organisational and legal building blocks and technical building blocks. The organisational and legal building blocks define issues typically done as human processes, while the technical building blocks define software systems or components. Both descriptions are generic, requirement descriptions. The building blocks are published as blueprint documents and will be supported by a catalogue of toolboxes containing the functionality implementations [47].

### 2.4 Brief state of the art of data spaces

In this brief section, we use the current data space concept defined in [57]. The main initiatives and specifications are the ones presented in the previous section, i.e., the IDSA reference architecture model [59], the GAIA-X association reference architecture model [60], and the Data Space Support Centre (DSSC) Blueprint 1.5 [47]. The IDSA model focuses on industrial data spaces that are meant to implement the needs of specific use cases. The GAIA-X model aims to build trustworthy data-sharing infrastructures, and the DSSC model's primary goal is to support the development of common European data spaces. The SIMPL project [5] is a European initiative to build reference implementations for data spaces, and it has published its own architecture model as well. The initiatives in Japan [62,63] and China [64] show that the concepts are spreading outside Europe.

Various reference implementations have been developed based on previous models. The most common are the IDSA Test Bed [65] and its certified implementations (VTT DIL, T-systems, TNO, Tekniker, …). Eclipse Tractus-X is an implementation of GAIA-X specification [66]. Eclipse data space components attempt to combine the ideas of IDSA and GAIA-X to a single implementations [67]. SIMPL Open is a test bed implementation of SIMPL components [68].

Data spaces have led to some commercial offerings. Sovity [69] and Data Space Europe [70] offer software solutions for setting up data spaces. There are also companies such as [14] that offer data management extensions for data space type of data sharing. Novatrust [71] is an example of consultancy services for data space operators.

Data Space Radar [72] is a service that collects data on space initiatives and developments of various life cycle stages. Smart Connected Supply Network [73] and Catena-X operated by Cosify [74] are examples of operational stages. Common European Data Spaces [21] are currently in the development stage, the mobility data space, the tourism data space, and the health data space are the most mature. Additionally, there are a huge number of data space experimentations in EU research projects.

## 3 Research Approach: Logical Functional Modelling

Logical functional modelling is a part of the requirement engineering in the typical system design process. It aims to specify the planned functionalities of the system as a pure function independently of any implementation alternatives or restrictions. The logical modelling ideas have existed since the 1970s when the structured design of software systems became a necessity due to the increasing complexity of the systems. Structured analysis [75] involved modelling techniques such as data flow, state transition, and entity-relationship diagrams to describe the logical composition of the essential system elements without implementation constraints. Object-oriented methods in the 1990s introduced new requirements that led to the development of the Unified Modelling Language, UML. The UML integrated the earlier methods and added new concepts such as class, activity, and component diagrams. The landmark paper of Kruchten [76] divided the system modelling into 4 + 1 views of the system architecture. The approach presented different architectural views for various needs and allowed system designers to focus on specific topics instead of modelling everything simultaneously. The four views are the logical view, which focuses on functionality as objects and object classes; the process view, focusing on concurrency and interaction; the physical view, which maps software onto hardware; and the deployment view, describing the static structure of the software system. The fifth view is the scenarios that integrate the other views with the use cases. Recently, the development has gone towards Architecture Description Languages such as ArchiMate, including enterprise and business modelling aspects of design [77].

The research presented in this paper is based on the constructive research approach [78, pp. 95–107] that aims to solve practical problems while producing academic theoretical contributions. In this work, we study the basic problem of trustworthy data sharing between legal entities that we extract from use cases and definitions presented in earlier research presented in Section 2. The logical functional architecture model has been created both using the earlier existing approaches and developing new descriptions of needed functionalities. The target has been to eliminate implementation dependencies and focus on logical behaviours.

The modelling approach chosen in this paper looks at the data space as a system. It starts by modelling the system’s use cases and translating them into an architecture model consisting of logical components. The use case part is based on the IEC62559 standard [79] that uses the UML use case model approach. To model the functionality of use cases, we used activity diagrams to present functional activity flows. Diagrams are supported with textual explanations. The logical architecture is modelled using UML component diagrams, but it must be noted that these components are independent of implementation. They present a logical collection of functionalities that may be implemented with several technologies and human operations.

The presented model has been validated by mapping its main concepts to existing data space specifications and models, i.e., IDSA reference architecture, GAIA-X reference architecture, and DSSC Blueprint.

## 4 Data Space Model

We propose the following definition of data space that is used as a basis of our models:

*Data space is a trustworthy infrastructure for secure and contract-based data exchange and its rights between identifiable legal entities that are members of data space and committed by a legal agreement to operational principles and rules of the data space*.

The purpose of this definition is to emphasise what data spaces do and the sources of trust, trustworthiness, and data sovereignty. Trustworthiness is a complex concept that also includes subjective aspects. In this definition, the trustworthy infrastructure refers to implementing data space functionalities using the principles described in subSection 2.2.4. Secure means protection against cyber threats and unintended exposure of data. As defined in subSection 2.2.1, data sovereignty requires transferring rights associated with data assets, identifying entities that own the rights, and committing to data sovereignty. The legal aspects require that data exchange is based on contracts between parties. Identification of parties requires that legal entities, i.e. companies, organisations, associations, and people, have verifiable identities given by identity providers and approved by data spaces.

### 4.1 Use case model for data space

**Fig. 4:**
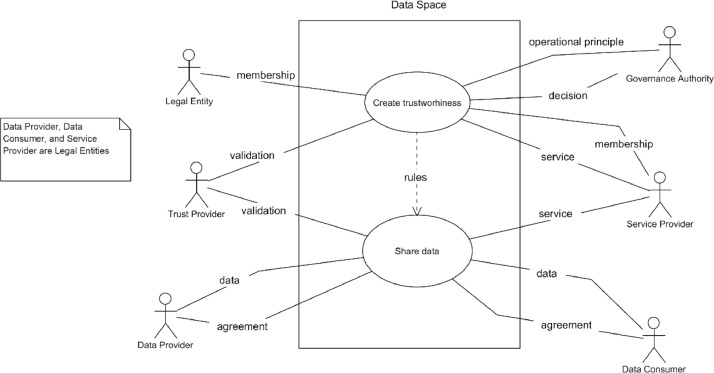
Basic use case of data space.

The top-level diagram of the basic use case of a data space is in Fig. 4. The purpose of the data space is to create added value for the users by sharing data using trustworthy infrastructure. This is divided into two main use cases: manage data space and share data. The actors in the use case are:

• *A user is a legal entity that can be a data provider, a data consumer,* or have both roles. The user either shares or receives data via the data space.
• *Governance authority*, which provides rules for data space operations and is responsible for running and managing data space. Governance authority can be a single legal entity that provides the whole data space as a service, or a set of legal entities that jointly make decisions about the data space.
• *Service Provider*, who provides the functionalities of the data space.
• *Trust Provider*, who provides the verification of the claims related to trustworthiness both in management and operational situations.

#### 4.1.1 Manage data space

**Fig. 5:**
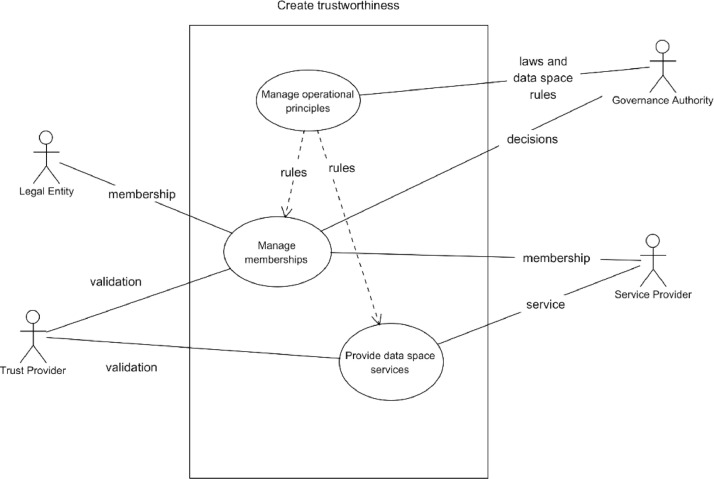
Manage data space use case diagram.

The Manage data space use case, presented in Fig. 5 is divided into three sub-use cases:

• the management of operational principles of data space, i.e. how the data space works,
• the management of the memberships, i.e. managing the collaboration network that can use data space services, and
• the provisioning of data space services, i.e. running the data space.

##### 4.1.1.1 Manage operation principles

In managing operation principles, the governance authority defines the rules for how the other two sub-use cases should be handled and includes these rules in the data space's governance framework. The data space's members commit to adhering to these rules by signing legally binding agreements.

Every data space can have its own rules and principles, and the governance authority can also decide the level at which they are defined. The rules specify how the main data space requirements concerning data sovereignty, trustworthiness, security, user rights, data space management, etc. should be implemented. The governance authority is also responsible for ensuring that the data space operates according to these rules.

Operational rules should be defined before data space is operational, but some rules define processes that need to be executed during the operational phase of a data space. They will create management data that needs to be maintained and updated by the data space governance authority or the service provider.

It is impossible to list all the possible principles of data space, even at a logical level. The following tables list examples of the main concepts and descriptions that should be covered. Table 2 is about collaboration network and membership issues, Table 3 deals with trustworthiness, and Table 4 with operational issues.

**Table 2:** Operational rules regarding memberships.

Concept	Description
Membership criteria	The requirements for a legal entity to join the data space.
Membership evaluation process	How members are evaluated during the joining process.
Termination of membership	What can cause a termination of the membership?
Member duties	What are the member's duties, such as payments and commitment to the rules?
Member rights	What can the member expect from the data space and its service providers?

**Table 3:** Operations principles regarding trustworthiness.

Concept	Description
Member identification	How members' identities are verified when a member takes action using the data space services.
Service trustworthiness	How services and service providers can be trusted. This can include restrictions and requirements such as certification and evaluation processes.
Security solutions	Communication and data must be handled securely.
Legal framework	What are the laws and regulations that need to be followed in operations?
Dispute management	How can disputes between members or members and operators be solved?

**Table 4:** Operational rules regarding running of data space.

Concept	Description
Data space purpose	What is the purpose of the data space and how does it affect the possible use of data space services?
Data sharing restrictions	Are there any data space-specific restrictions on data sharing?
Data space service providers	Data space should have descriptions of its services and service providers, their trustworthiness, and operation monitoring activities.
Data space collaborations	It should describe whether the data space collaborates with other data spaces or has data solutions.
Data sharing contracts	Are there contract templates or models primarily enforced by a data space? Are there specific contract conditions that data space supports? Are the particular constraints to contracts?

##### 4.1.1.2 Manage memberships

Management of data space memberships deals with joining and leaving the data space and managing the data space collaborations. Joining and leaving involves both users and service providers, but their processes may differ. Data space collaborations involve at least governance authority. Still, they may also involve service providers and even members, depending on the level of detail at which the collaboration will be set up.

**Fig. 6:**
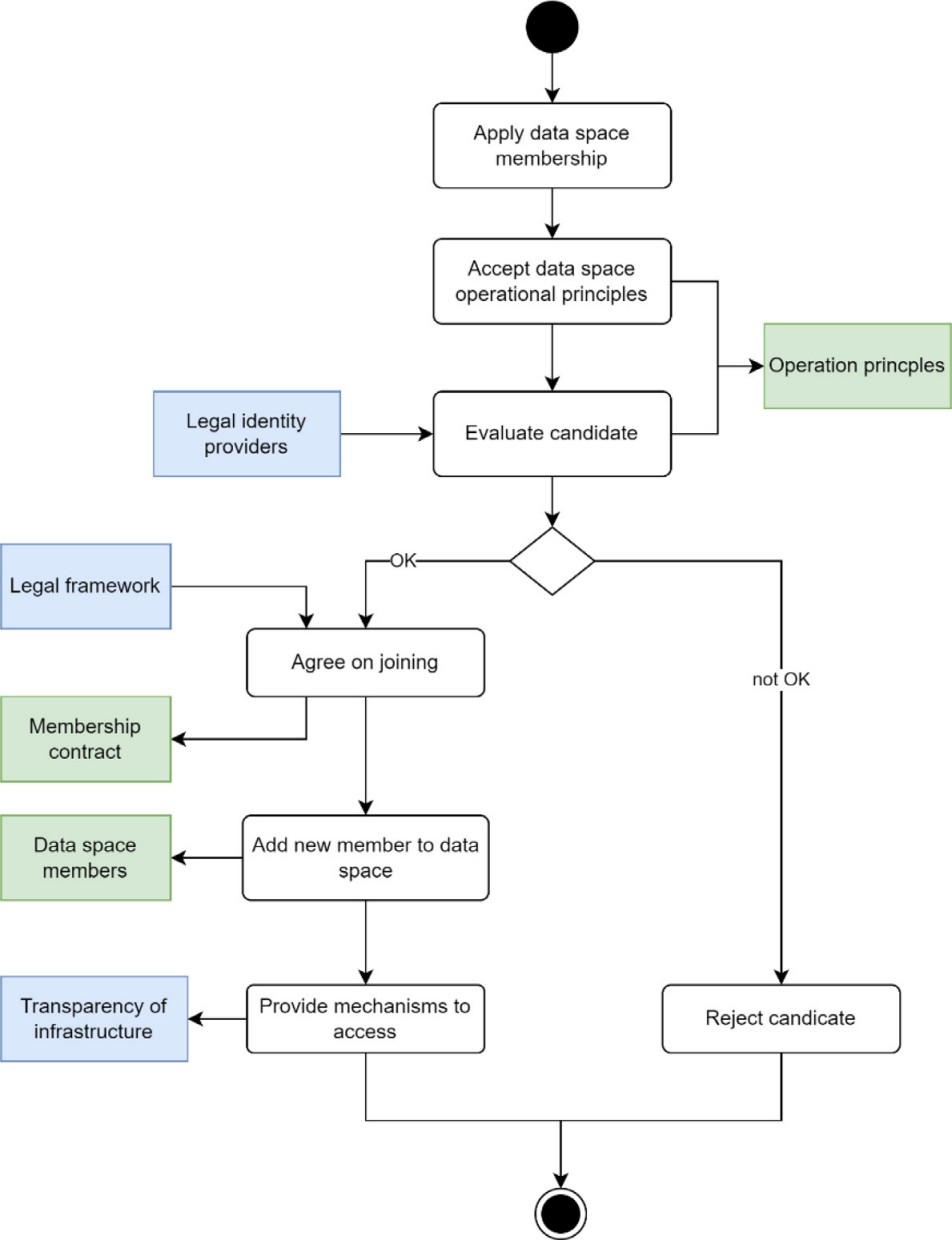
The process of adding members to a data space is as follows.

The steps of the onboarding process, depicted in Fig. 6 are the application of membership, commitment to data space principles and rules, evaluation of the candidate, and activation of membership that involves mutual agreement, adding legal entity to data space members, and providing access mechanisms to data space services. Naturally, if candidate evaluation fails, membership is rejected. It may be needed by data space rules that the claims presented by applicants are verified by the trust providers. The final decisions depend on the governance authority, but the processes can be automated.

As seen from the Fig. 6, the outcomes of the process are the membership contract, adding an entity to the data space members, and contribution to the transparency of the infrastructure. **The membership contract should be legally binding** between the governance authority and the joining entity. Its purpose is to prove the entity’s commitment to data space’s operational principles and data space’s intention to provide useful services to the entity. **Data space members** is a list maintained by the data space that contains the key information of its members. The main purposes are membership management and access control. Contribution to infrastructure transparency comes from the requirement that the mechanisms used in operations in the data space must be trustworthy. Therefore, the data space must be sure that the mechanisms used by a new member are trustworthy.

**Fig. 7:**
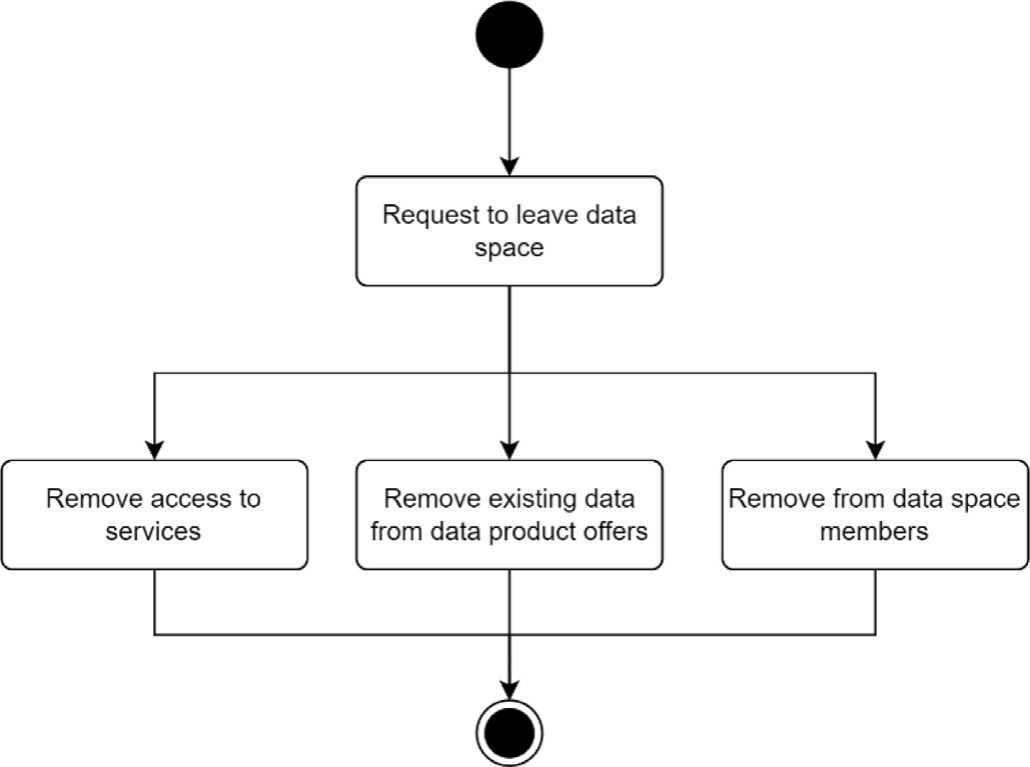
Process of removing a member from a data space.

The process for removing members from a data space is depicted in Fig. 7. Removal of a member can be caused by infringement of the data space rules or by request from the member itself. The reason for removing a member depends on the data space governance rules, which should be described in the operational principles. The actual removal consists of three actions. Removal of access to services happens when the access mechanism is invalidated. The data related to the entity must be removed from the data space members list. Finally, the data product offers listed in the data space product offer list must be removed.

##### 4.1.1.3 Provisioning of data space functionalities

In the provisioning services sub-use case, the service providers provide the needed functionalities of the data space. This involves the implementation of the functionality, acceptance of the functionality by the governance authority, and provisioning and maintaining the functionality for data space users.

Implementation of the functionality depends on the chosen architecture and specification of the data space. As the data space is a software system, the most common approach is to develop software services and deploy them into the service provider's system. However, data spaces can be implemented in any software architecture.

Data space functionalities must be trustworthy, and trustworthiness must be transparent. Governance authority must guarantee this to data space users; therefore, the governance authority must accept all the functionalities**.** How acceptance is given and how the quality and trustworthiness of functionalities are evaluated and validated depends on the data space's operational principles. Possible approaches are using open-source implementations and certification by an independent certification organisation.

Provisioning and maintaining functionalities are done like in any secure software system. The key requirements are related to **the identifiability of implementations of functionalities and their immutability**. The trustworthiness principle requires the user to ensure that the operations are carried out with trustworthy implementations and that the messaging between those functionalities is secure. This means that all the **messages must be identifiable** and **not accessible by third parties**, all functionalities must be identifiable, and it must be possible to verify that they have not been modified by any means.

#### 4.1.2 Sharing data

The data-sharing approach needs to have ways:

• to represent data as a product, whose existence can be published to the possible users or partner network,
• to negotiate and execute a contract between data sharing parties that defines what is shared, what rights are transferred, how the contract and data sharing are executed, how the contract execution is monitored, and how disputes are solved,
• to share the data itself.

**Fig. 8:**
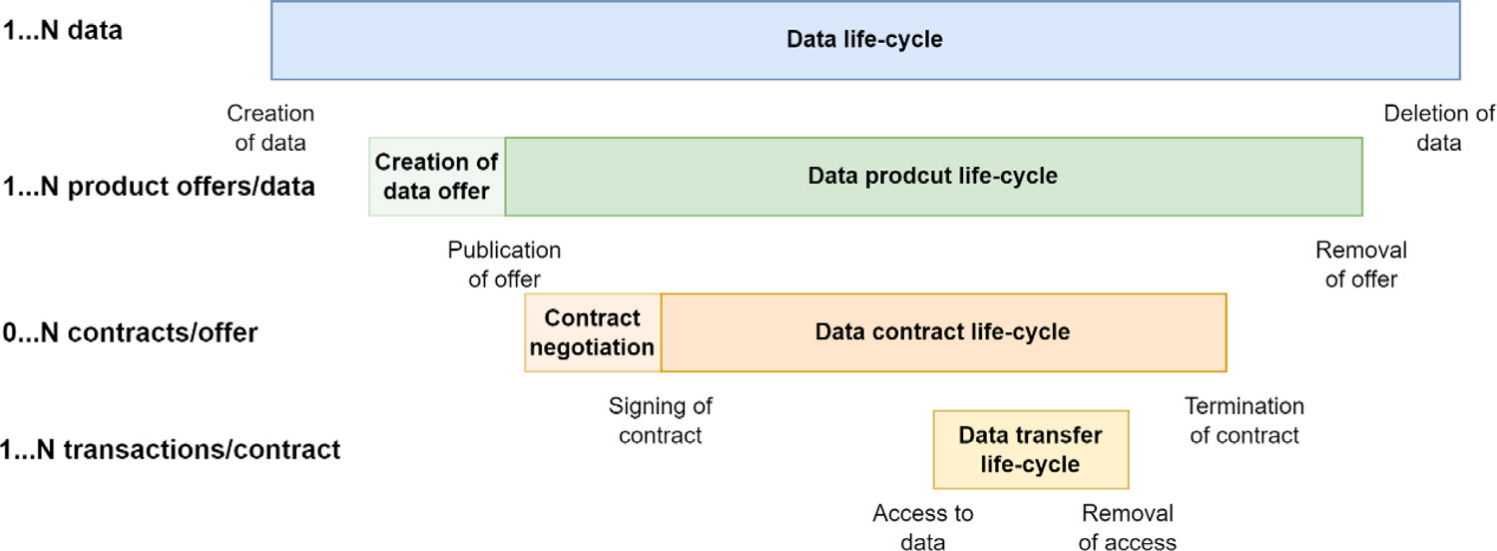
Lifecycles in data sharing.

The life-cycle relation of these functionalities is depicted in Fig. 8. Data itself exists before the data product can be created and can remain exist after the data products based on it have been deleted. Similarly, the data contact cannot be made before the data product exists, and the data product should exist until the contract is terminated. In a data space, the data transfer life cycle is part of the contract. The purpose of the figure is to show only the time and number of dependencies of data transfer, data sharing contract, and data product and that they are dependent on the data life-cycle in the sense that data or data service must exist during these other processes.

**Fig. 9:**
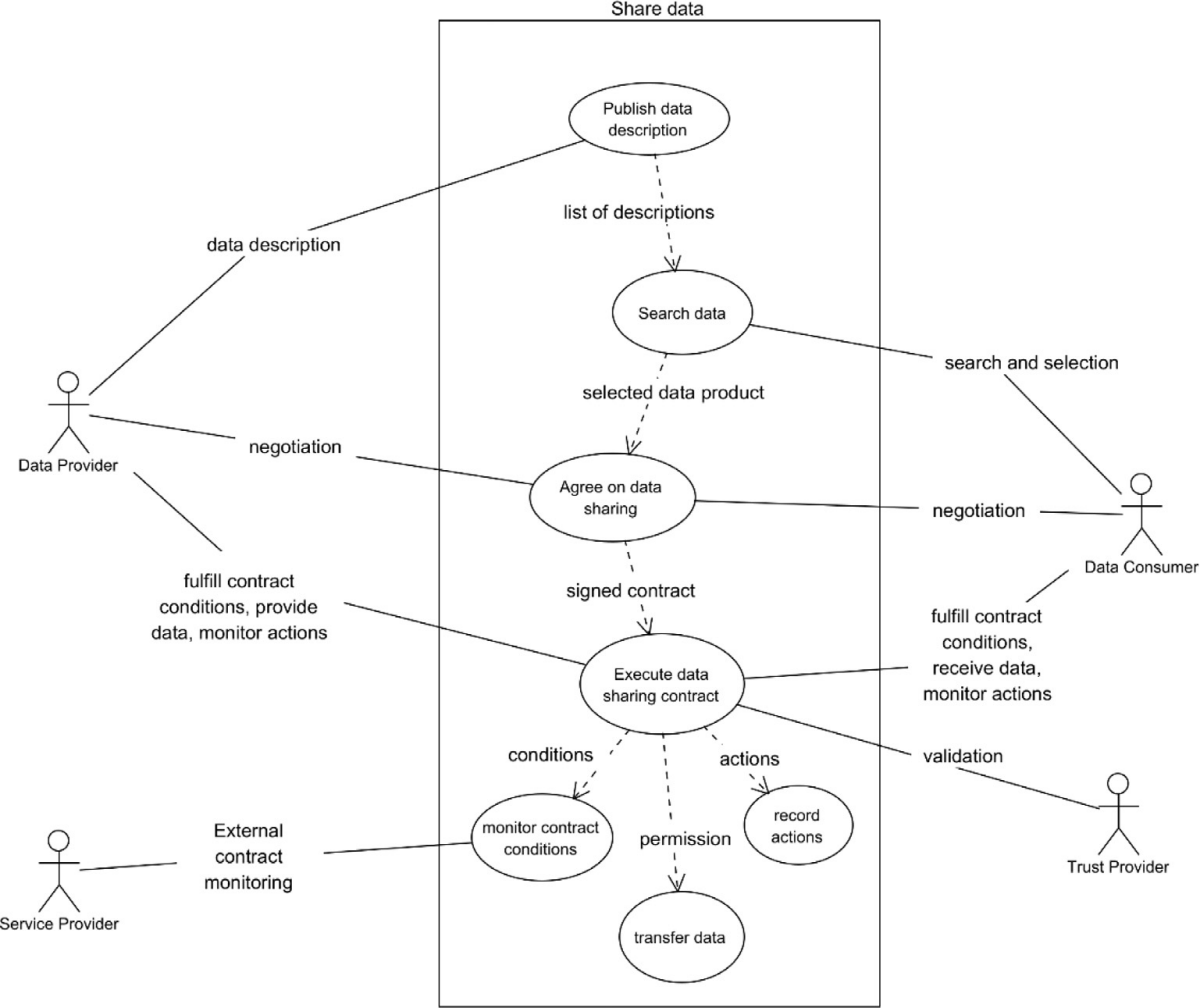
Share data use case diagram.

Contract-based sharing of data between two legal entities, the data provider and the data consumer, as presented in Fig. 9, is the main use case in the data space. The data sharing consists of three main phases:

• Publishing and searching data.
• Negotiation of the data sharing contract between the data provider and data consumer, and
• Execution of the contract so that all contract conditions are implemented and validated, data is transferred as agreed between the data provider and data consumer systems, contract execution is monitored, and actions are recorded in a secure and persistent way.

All the operations must be done in a secure and trustworthy environment and using secure and trustworthy functionalities.

The actors in data sharing are the data provider, the data consumer, and a service provider if the data sharing contract has conditions that need to be implemented and validated by third parties.

##### 4.1.2.1 Publishing data descriptions

**Fig. 10:**
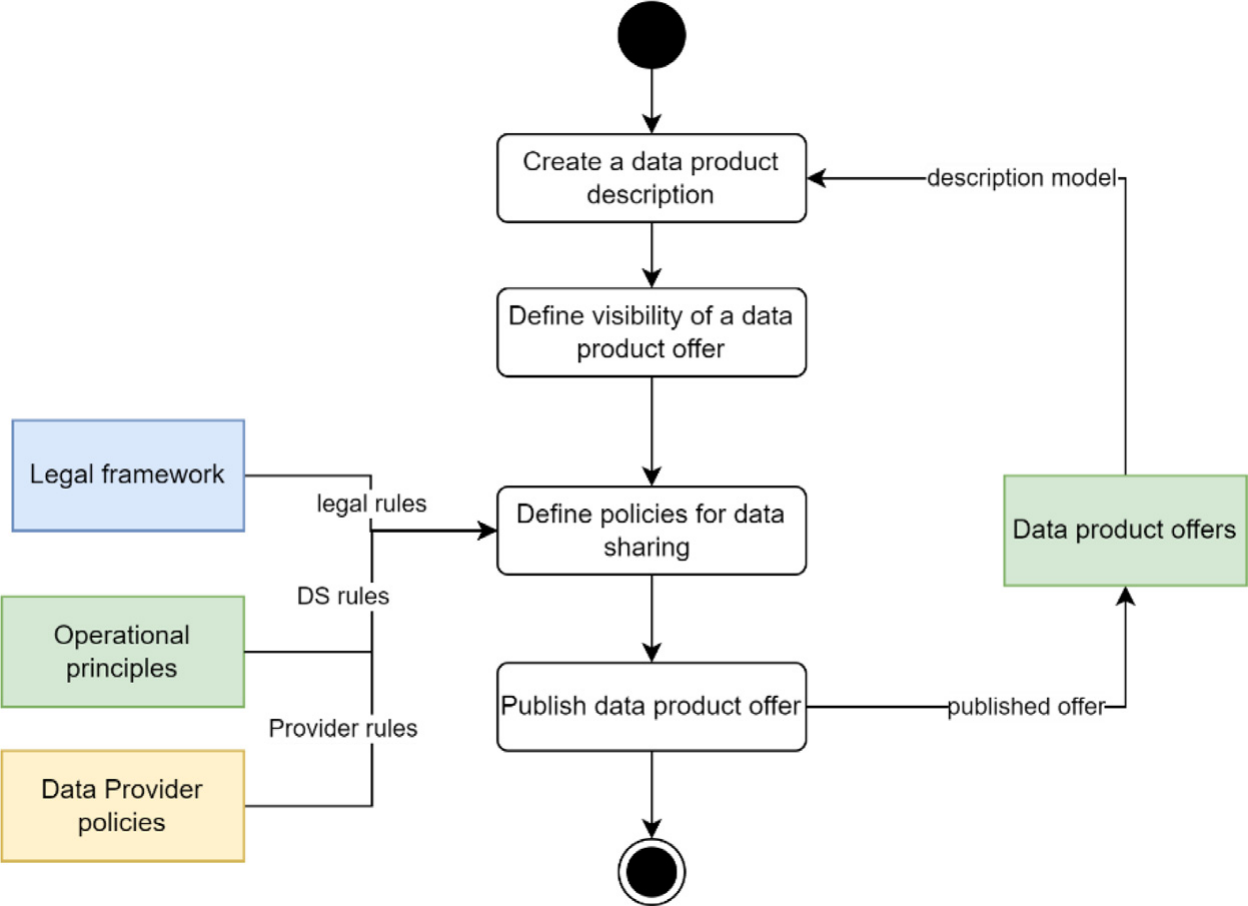
Publishing data products.

The first sub-use case in data sharing is that the data provider publishes data descriptions or data products so that possible data consumers can search, evaluate, and request the data. The process is described in Fig. 10. It involves the identification of data to be shared and how it is shared, the definition of who can see the data offering, the definition of conditions on which the data and its rights can be shared, and the actual publishing of the data product offer so that eligible potential consumers can see that it is available.

The outcome of the process is a **data product offer**. The offer must include at least the following:

• Who is sharing and owning the rights to data?
• The description of the data,
• What are the data rights to be shared?
• How will the data be shared?
• What are the conditions for sharing the data? These can include, for example:
  ○ Legal constraints, e.g., restrictions to shared data outside the EU or the country, such as criminal data.
  ○ Specific rules in the operational principles of data spaces, i.e., a data space may exist for a specific purpose, and data to be shared using it may be limited to that purpose.
  ○ Specific conditions data providers require, such as pricing, usage constraints, etc.

Data providers must have complete control over their data, so they must be able to control who sees what their data offers. Therefore, it must be possible to define the **visibility of data offers**. The visibility can be determined through inclusion, i.e. defining who can see the offers, or by exclusion, i.e. who cannot see the offers. Various technical means can be used such as explicit lists, characteristics of searching entities, distribution of pre-defined certificates, etc. It is up to a data space to define what mechanisms can be used, if any. The value of visibility control is that it allows for defining smaller dynamic collaboration networks inside the data space, which differs from what the data space membership does.

The data-sharing conditions are part of the **initial contract proposal** from the data provider to possible data consumers. It should define what should happen before, during, and after the data transfer. The initial contract proposal can be fixed or negotiable.

##### 4.1.2.2 Searching data

**Fig. 11:**
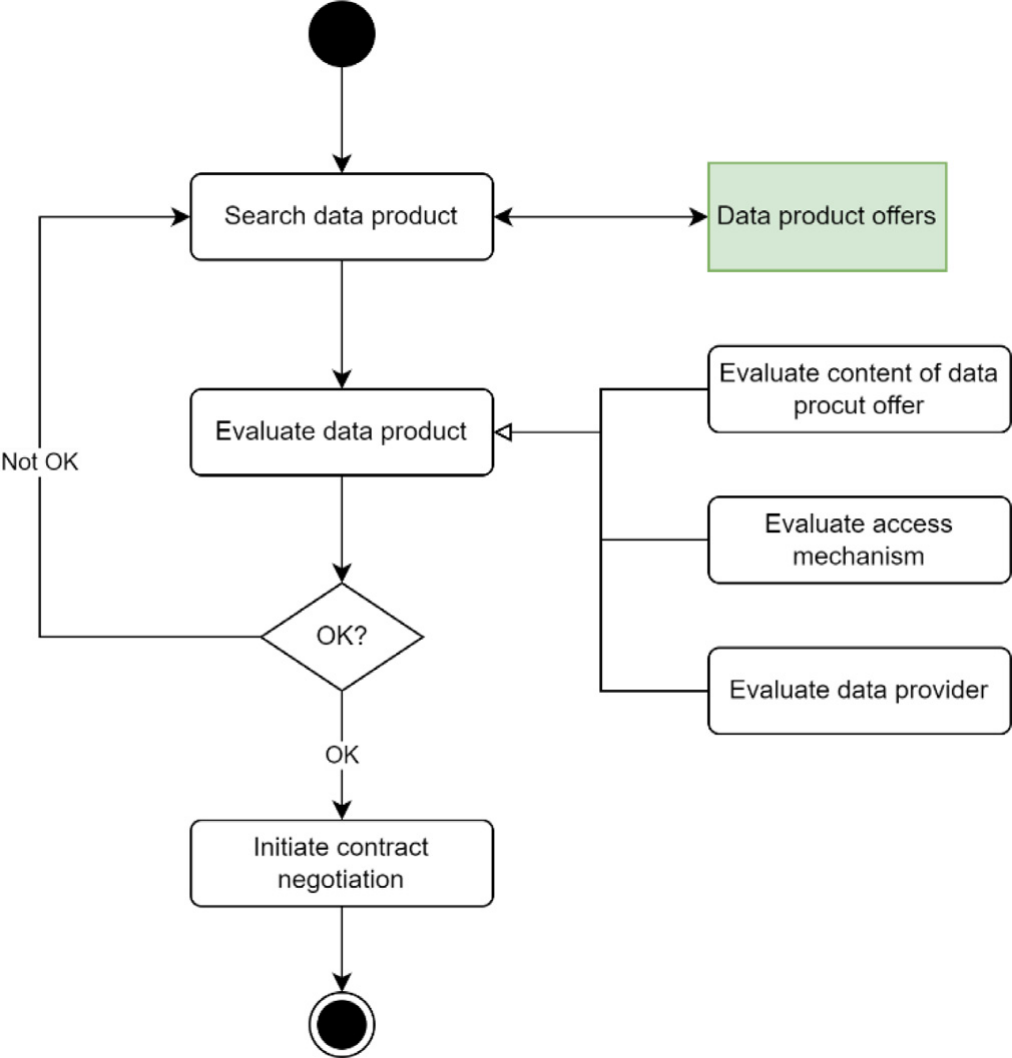
Searching and evaluating a data product offer.

The second sub-use case is the possible data consumer searches data from the data space. The process is described in Fig. 11. The consumer must be able to browse through or query the data product offers that exist in the data space and are visible to it. When interesting data product offers are found, the data consumer must be able to evaluate the data offer, i.e.,

• to evaluate the content of the data offer to understand that it fits the intended purpose,
• to evaluate that it is capable of receiving the data from the provider, and
• to evaluate the data provider is such that it wants and can do business with it.

These are the consumer's requirements for the data product offer descriptions.

If the result of the evaluation is that the data consumer wants to access and use the data, it must start the contract negotiation with the data provider. The data provider must have the possibility to decline the negotiations.

##### 4.1.2.3 Agreeing on data sharing conditions

This sub-use case aims to create a data-sharing contract based on the initial contract proposal. The data-sharing contract is a set of contract conditions that are implemented during the execution of the contract. The negotiation process is basically like any business negotiation between two parties. Both parties must be able to propose changes or additions to contract data or conditions and either reject or accept them. The process continues until a common agreement is reached or either party wants to end the process.

The contract conditions should describe:

• What has been agreed.
• If there are actions in the condition, how and who implements them.
• What is the criterion for accepting the action, and
• How the contract condition is validated.

Both parties must agree to the agreement of the contract, and it must be legally binding, which legally means under the rules of both countries of the parties. Data space principles determine the acceptance process. It can be, for example, a signed document or accepted in a data space service software. The final contract must be delivered to both parties and kept safe from possible disputes.

##### 4.1.2.4 Executing data sharing contract

**Fig. 12:**
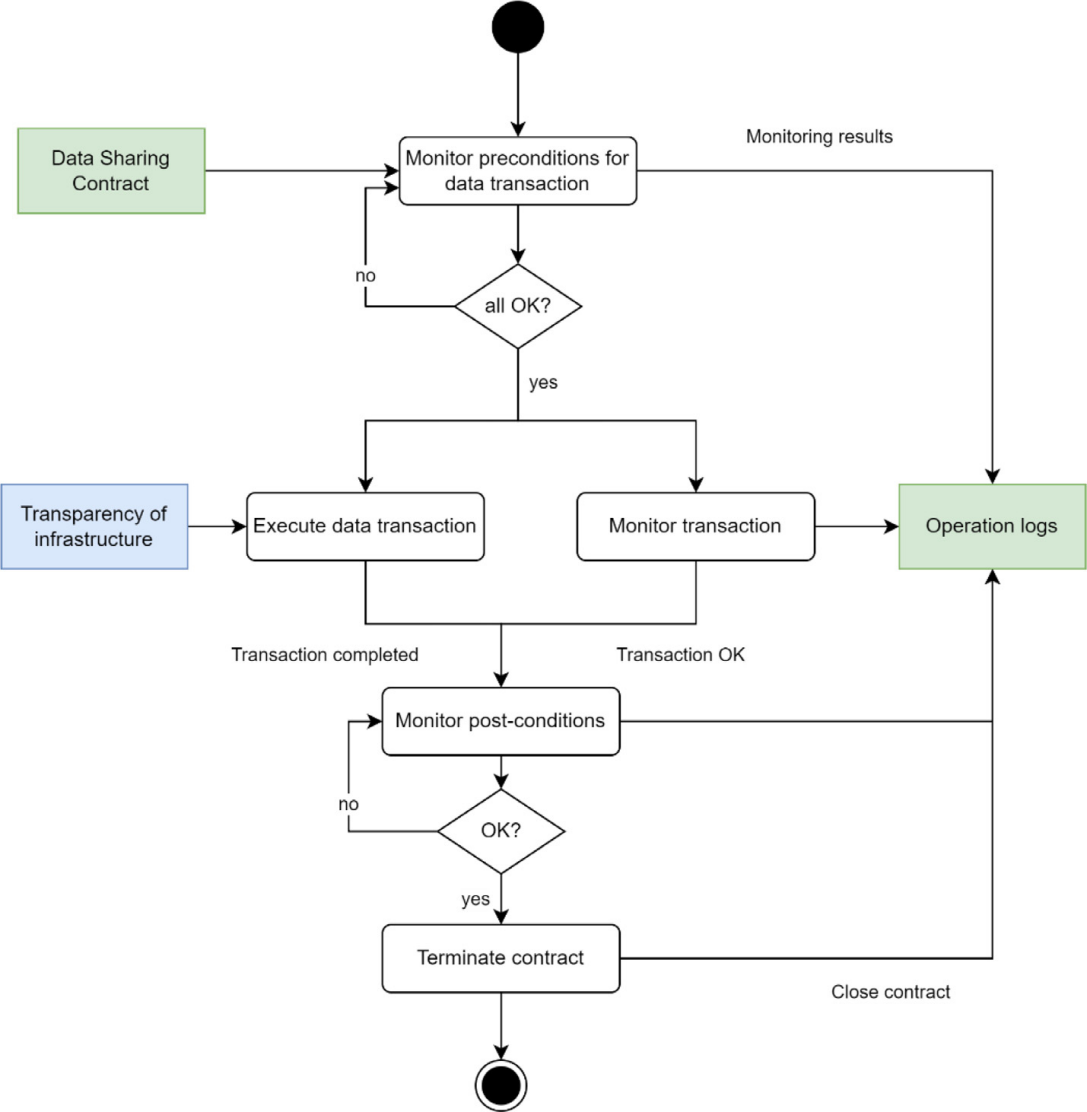
Exchanging data products.

Execution of data sharing contract is a process where each contract condition is implemented, evaluated, and logged according to the order defined in the contract (Fig. 12). The process starts with conditions to be fulfilled before the actual data transfer occurs. Such conditions can be related to setting up the data sharing capabilities, logging actions, possible prepayments, etc.

When all pre-conditions have been fulfilled, the data transfer phase may start according to the definitions of the contract. During the data transfer, the contract conditions, such as the time when transfer is allowed, the number of transfers, etc., must be monitored.

After data has been transferred, the post-condition needs to be evaluated and if everything is OK, the contract will be terminated.

During the contract execution, both parties must be notified of all possible abnormalities or infringements, and it must be possible for either party to stop the contract execution and data transfer at any time.

As the contract can include conditions that are very likely dependent on third parties such as payments and banks, there must be interfaces to those that allow the implementation of those conditions externally to the data space itself.

### 4.2 Environment model

When we think of the data space as a system at the logical level, we must separate the system from its environment. Then the functionalities described in use case models must be converted to architectural modules of the systems and descriptions of their purposes.

**Fig. 13:**
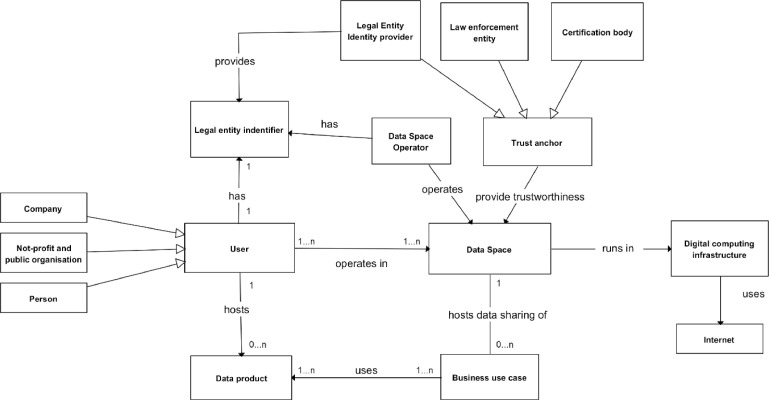
Data space environment model.

The environment model of a data space is described in Fig. 13. A data space system is used by users who can be companies, non-profits, public organisations, or persons. Still, they must be real entities with legal entity identifiers provided by legal entity identifier providers, typically government organisations of a country where the user is. Users interact directly with the data space system via a human-computer interface, a computer interface, or both. Users also host data products used by business use cases, whose data exchange is done via the data space system using APIs and software services.

The data space is operated by the data space operator, who must also be a legal entity. There must be a management interface for the operator. The trustworthiness of the data space comes from trust anchors that are legal entity identity providers for users’ trustworthiness, law enforcement entities such as the judiciary, judges, police for handling disputes, and certification bodies for guaranteeing the quality and security of system resources. Trustworthiness can be injected into a data space by providing immutable certificates as part of services and communication or by having loosely coupled processes that ensure the respect of data sovereignty and user rights. If we are talking about the digital system, it runs on digital computing infrastructure, i.e. computers, servers, or cloud, and is based on communication via the Internet.

### 4.3 Logical architecture model

**Fig. 14:**
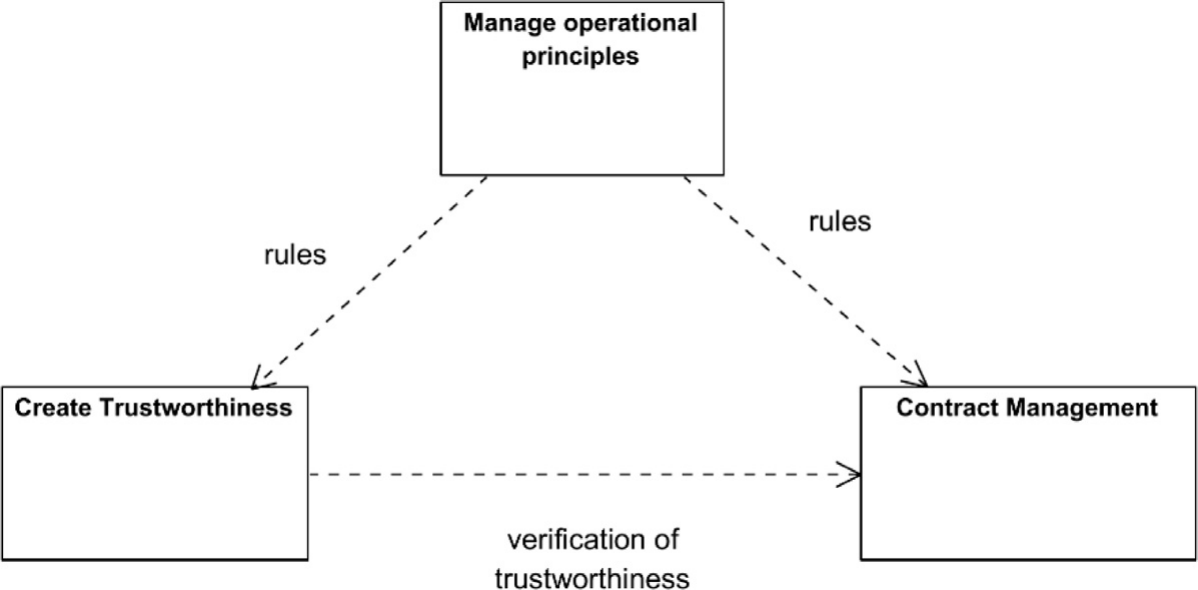
Main logical components of a data space system.

The main components of a data space system are the management of operational principles, the creation of trustworthiness and contract management (Fig. 14). Operational principles define the rules of a data space. The trustworthiness component contains functions needed to make data space a safe, secure, and trustworthy environment for a company to exchange confidential information. The contracts management part includes the services needed to implement the contract processes that start with the creation of an initial contract proposal as a part of the data product offering, continue with the contract negotiation, and finish with the contract execution and termination.

The logical architecture does not describe where the actual functionalities are deployed or whether they are deployed as a single or distributed functionality. In some parts, it is not even specified whether the described functionality is completely part of the data space system or the user’s own system. Examples of such functionalities are data product offerings and contracts. With a big company with hundreds of data offerings and contracts, it would be more efficient and secure to build our own implementations for managing them.

#### 4.3.1 Management of operational principles

**Fig. 15:**
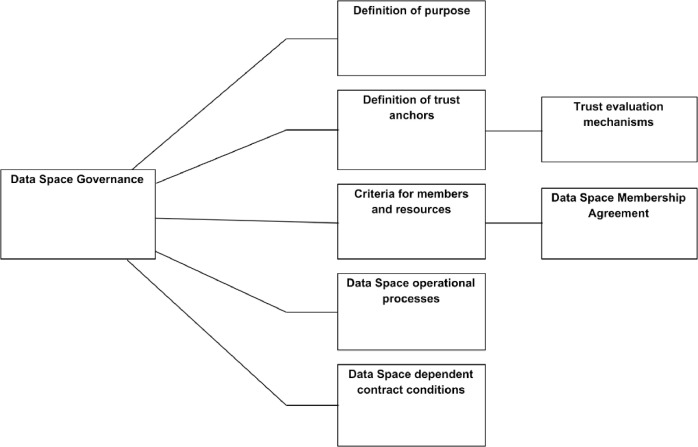
Management of operational principles.

The main component in managing operational principles is data space governance. At this logical level, the model combines various aspects of governance to make the model generic. Splitting governance into smaller details would prefer some implementation styles of data space over others. As described in Fig. 15 the data space governance components produce the following outputs:

• A definition of the purpose of the data space is needed to understand why the data space is defined as it is. Purpose also helps companies evaluate the various data spaces and their offering if made visible to them.
• Definition of trust anchors that are used in the data space. Related to this are also components for using the trust anchors when data space is operational.
• Criteria for members of data spaces and resources used in a data space. Even though a data space concept is meant to promote data sharing, the individual data spaces can define criteria for possible members and decide members by accepted members. The criteria are formalised as data space membership agreements, where each member commits to the data space rules, and the data space commits to provide the agreed services for the users.
• Definition of operation processes. This translates to implementing data space resources and how they are used. Examples of processes are member onboarding, resource evaluation, etc.
• Definition of data sharing contract conditions dependent on data space itself.

Management of operational principles is the task of the data space operator. The data space operator or governance, however, can have various forms outside this paper's scope. The only limitation is that a legal entity or entities must be responsible for implementing the operations.

#### 4.3.2 Creation of trustworthiness

**Fig. 16:**
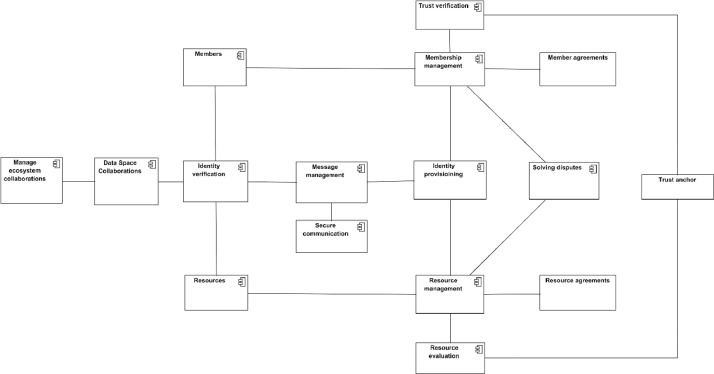
Components for the creation of trustworthiness.

Creation of trustworthiness is based on three pillars in data space: Knowing who and what you are interacting with, knowing that the parties are committed to following the same rules and regulations and that there is a legal system protecting your interests and transparency of the implementations of the functionalities that are used. These three pillars need the logical components presented in Fig. 16.

Knowing who are valid members requires a list of **members and data to evaluate the members and collaborate with them**. The Members component must be able to serve the data requests addressed to it according to the rules of operational principles. To become a member, a component called Membership Management must handle the applications, membership evaluation, member monitoring, and possible leaving of a data space. **The trust verification component is related to membership management, and it is needed to verify** using the trust anchors that the claims presented by the member candidates are true. The commitment to common data space rules is ensured by the data space Membership agreement, which is a result of joining the data space. The data space must also have a component for **Solving disputes** in case infringements of agreements or contracts have been detected. Implementing this component may involve authorities, lawyers, etc., as a possible set of disputes can be very broad.

To know what valid data space resources are, there must be a component called **Resources**, which is a list of accepted resources and how they can be accessed. Further, a component called **Resource management** is needed to implement acceptance and maintenance processes. The resource providers must commit to the data space rules similarly to members with a legal **Resource agreement**. The **Resource evaluation** component is needed to ensure the security, trustworthiness, and transparency of resources, and trust anchors should be used to ensure trustworthiness.

The data space components communicate using messages. **Message management** component is for ensuring that messages are transferred securely, immutable, and that they come from where they claim to be and that they are related to the data space operations. **The security communication component** is an obvious part of this. Without security, no confidential data would be shared, and no data-sharing contracts would be negotiated. There must be means to implement needed cyber-security solutions, secure communication channels, etc.

A key part of the data space is that all members, resources, and messages can be identified to belong to the data space operation. The **identity provisioning** component gives identities and **Identity verification** checks that they are correct when members and resources communicate using messages. It is important to note that identity refers to data space, but the process of accepting members or resources must involve the verification of the original real-world identity from the trust anchor. Maintaining this chain of trust is an essential requirement for the data space.

The data spaces do not necessarily exist in isolation. There may be a need for data space to interoperate with another data space. Meaning that a data space member wants to access data available in another data space from its member. The motivation for such collaborations is that data space will provide means for secure contract-based data transfer with entities that have passed the membership criteria and evaluation processes, i.e. they have been proven trustworthy in their own data spaces. Such exploitation needs to be approved by the data space operator, so there is a need for a component called Manage Ecosystem Collaborations, and there is a need for a component called Data Space Collaborations to enable the collaborations on an operational level. The actual implementations of these components depend heavily on the chosen interoperability models and data spaces involved.

The data space operator should control all the components related to trustworthiness; however, for example, solving disputes easily expands to third parties, and secure communication has parts from telecom or Internet operators.

#### 4.3.3 Contract management

**Fig. 17:**
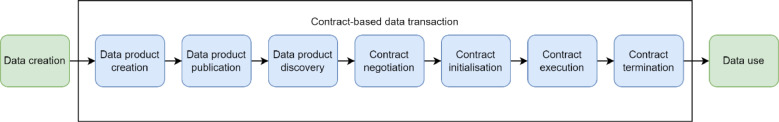
Contract-based data transaction process.

The data space implements a contract-based data transaction process described in Fig. 17. The process needs data that need to be productised, published, and discovered before the actual contract negotiations, initialisation, execution, and termination can take place. The final purpose is to use the data by the receiving party.

**Fig. 18:**
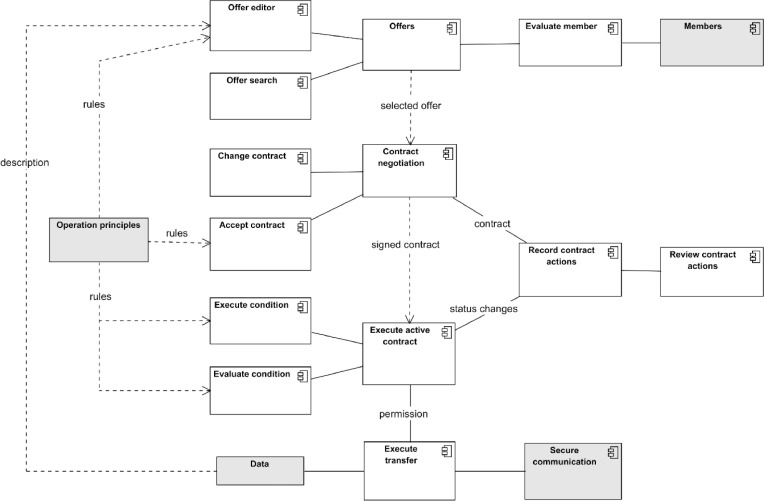
Components for management of data sharing contracts.

The logical architecture of contract-based data transaction has three main components: Offers, Contract Negotiation, and Execution of Active Contract as depicted in Fig. 18. From a user perspective, these components are the essential functionalities of data space, but all these components need supporting components to enable their use.

**Offers:** Offers aim to make the data product offers created by data providers visible to data space members according to visibility rules defined by data providers. Making visible means that data consumers can search for offers and evaluate them to select the ones they see feasible for their purposes.

• The **offer editor** component is for creating, reading, updating, and deleting the data product offered by the data provider. The core of the data product offer is the existence of data to be shared. The content of the data and the rights to the data need to be described in the offer. The offer must also comply with the rules of the data space.
• **Search data** component is for browsing or querying the available data products. In addition to the preparation of search criteria, it must support the presentation of the data product offer data. This component's level of intelligence is a key success factor of a data space. This is naturally meant for a data consumer.
• **Evaluate data offer** component is needed by data consumer to analyse whether the data product and its provider are suitable for the intended use. Therefore, this component needs access to both Offers and Members data. The evaluation criteria and methods depend on the data consumer.

**Contract Negotiation:** The purpose of Contract Negotiation is to enable the data provider and data consumer to negotiate the contract for the selected data product. The data product offer should contain the contract proposal with the contract content description, i.e., what and how data is shared, what usage and holder rights are transferred, and the other descriptions of contract conditions and their implementation and evaluation methods. The contract negotiation ends either with the acceptance or signing of the contract, which transforms the contract into an active contract and initiates the recording of the contract actions or the rejection of the contract.

• **Change contract** component is needed by both parties in the negotiation for reviewing, editing, removing, adding, and accepting or rejecting condition proposals.
• **Accept contract** component provides means to make a legally binding agreement between parties.

**Fig. 19:**
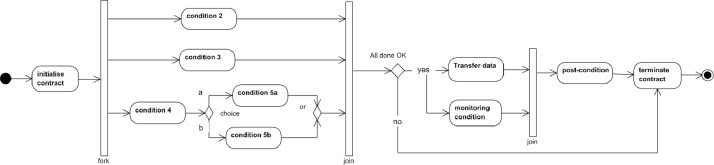
Example of contract condition graph.

**Execute Active Contract:** The component's purpose is to control the data-sharing process according to the conditions defined in the signed contract. Contract conditions can be presented as a graph in Fig. 19. Conditions can be divided into three phases. Pre-transfer conditions must be implemented before the data transfer. During the transfer, conditions must be active when the transfer is active. Post-transfer conditions must be fulfilled after the transfer. The process ends with contract termination. Inside the graph, there can be parallel or sequential conditions and different types of control structures defining the order of conditions. Execution active contract component must implement the graph and activate the condition implementation and evaluation components accordingly, and report the actions to the Record actions component.

• **Execute condition** components are needed for implementing the defined conditions. There will be many types of contract conditions, and they will be diverse. Some may be implemented with simple automatic checks, while others involve complex human processes.
• **Evaluate the condition components** needed for evaluation of the condition implementation results. Based on the result and condition graph, the execution controller does what is described.

**Execute transfer:** The purpose of this component is to enable communication between the data consumer and the data provider during the data transfer. It should support the different data services and their access methods. Using the secure communication component should also protect the data when it is transferred on the Internet.

**Record Contract Actions:** The component's purpose is to serve as an immutable storage of contract actions as evidence of contract execution.

**Review Contract Action:** The component's purpose is to provide access to contract records. It should be accessible by both data providers and consumers, as well as by third parties defined in operational principles, especially during conflict resolutions.

## 5 Comparison to Implementation Architectures

This chapter compares the logical architecture with IDSA, GAIA-X and Data Space Support Centre (DSSC) specifications. Direct comparisons are impossible as all these alternatives have different abstraction levels and views that are not fully compatible with the logical architecture. IDSA and GAIA-X are reference architecture models with close links to some implementation technologies and architecture principles. The DSSC model is a generic framework that looks at data spaces from a wide angle. It focuses mainly on the development of data spaces and covers business, organisational, legal, and co-creation perspectives that are outside the scope of the logical model. We have divided the comparison into three groups:

• Data space management issues that consist of governance, operations and data space services.
• Collaboration network issues that consist of the data space member topic and the data offers.
• Contract-based data sharing consists of contract negotiation, execution, and conflict issues.

This comparison aims to show how the current specifications map with our model and validate that the model describes what it is supposed to represent.

IDSA reference architecture consists of five layers: business layer, functional layer, information layer, process layer, and system layer. The primary reference in the comparison below will be done with the system layer as it defines the implementation components, but as the logical architecture components are implementation-independent, the relation to other layers’ content is also given when they address the components of our model.

GAIA-X architecture document is divided into ecosystem description, conceptual model, self-description definitions, operating model, federation services, and example use cases. The comparison is done mainly with federation services, but the conceptual model, self-description definitions and operating model address some components, too.

DSSC blueprint 1.5 divides the data space model into two categories of building blocks: business and organisational building blocks and technical building blocks. DSSC building blocks are descriptions rather than specifications. In the comparison, we provide the related building blocks. DSSC aims to develop a toolbox that contains alternative implementations of the building blocks, but that was not available when this was written.

Aas we can see from Table 5 all the main features of data spaces are covered in all approaches. The main difference is that in IDSA, GAIA-X, and DSSC the functionalities related to the user’s process, such as data product offer creation and management of the user’s data sharing contracts, have been excluded. The systems only provide an interface to manage the data products or contracts used in the data space processes.

**Table 5:** Comparison between logical architecture, IDSA reference architecture 4, GAIA-X architecture document 22.04 release and DSSC Blueprint 1.5.

Features	Logical architecture	IDSA reference architecture model 4.0	GAIA-X architecture document 22.04 release	DSSC Blueprint 1.5
Governance and operations	Operation principles: purpose, trust anchors, member and resource criteria, processes, specific contract conditions	The governance perspective defines models on how things should be done, which the IDSA Support Organisation implements. (IDSA-SO)	Participants. Management plane and federation services. GAIA-X registry as a source of trust. Possibility for autonomous organisation.	Organisational Form and Governance Authority. Contractual Framework
Data Space Services	Management of operational principles, creating trustworthiness, and contract-based data sharing.	Metadata broker, Dynamic Attribute Provisioning Service, Connector, Clearing house. Certificate authorities (CA). Resource acceptance by IDSA SO.	Identity and access management, data exchange services, portals and APIS, compliance services, catalogue services. GAIA-X Label issuer. Catalogue	Federation Services. Participant Agent Services
Data Space Members and collaboration network	The on-boarding process with the application, partner evaluation, and adding to the member list. Member monitoring and removing process.	On-boarding process by IDSA SO. ParIS: Partner Information Registry.	Catalogue with participant self-description that GAIA-X Trust Framework validates. Notarisation, Service, and Credential Storage.	Participation Management (Data Sovereignty and Trust pillar)
Data Offers	Offer list. Offer editor, offer search, and offer evaluation functionalities.	Metadata Broker. Publish and search APIs.	Catalogues with self-description graphs including claims that can be queried across catalogue boundaries.	Data Product, Data, Services and Offerings Description, and Publication and Discovery
Contract negotiations	Contract negotiation	Data space connector. Contract templates with parameters	Service Agreement Service	Participant agent services: Contact negotiation.
Contract execution	Execution of active contract	Data space connector. Policy enforcement services.	Smart contracts. Contract Agreement Service.	Participant agent services: Transfer process.
Conflict support	Record and Review Contract Action	Clearing house	Contract logging service	–

The logical architecture provides only a list of issues that must be implemented in the governance process. IDSA RAM 4 describes a traditional organisation-centric governance model, while GAIA-X has a collaborative model, where the operations can even be built as a distributed autonomous organisation (DAO). In this sense, the logical architecture model is more generic.

In the contract-based data transaction part, both IDSA and GAIA-X emphasise the need to enforce the contract conditions automatically using data space services. The need for other options, i.e. external or even human interaction-based contract implementations, is recognised, but the bias towards smart contracts is obvious. The support for handling disputes is very limited in all the models, as the processes are very complex too.

## 6 Discussion

The logical architecture model presented is based on the generic use cases of a data space that is the contract-based data-sharing between two parties in a trustworthy environment. The main objective in creating this model has been to raise the abstraction level of data space modelling above the existing reference architecture models and functional descriptions that use implementation technology-based concepts in their definitions. Such technical concepts define the implementations and limit the freedom to think of all the possible alternatives that might exist. Secondly, the conceptual models describe the data space concepts and their relationships but do not describe what the data space is supposed to do. The logical architecture takes a step towards explaining what data spaces are for, and the logical functional structure of the system, thereby providing a better starting point for the design of data spaces.

This paper focuses on modelling the core features of data space and explaining with simple models what a data space is. The presented models aim to be the first documents that a person who wants to be familiar with the data space concept should read. We have excluded many topics, e.g., in the DSSC Blueprint, such as how to set up the data space for some business use case purposes. These possible value-added services can be built on top of the data space or integrated as a data space service, as well as how business use cases should be operated, etc. We see the data spaces as an extension of the entity’s data management and not as a more complete decentralised or distributed data management approach. When a data asset is shared, it is duplicated, and both the original and the new data assets have their own data holder’s rights associated with them, and they continue their existence as separate objects. How data space is integrated or connected to data management is important to know when data spaces are created and operated, but this widens the scope to topics that, in our opinion, are not the core of data spaces anymore.

Core components in our model are contract-based data sharing and management of collaboration networks. Contract-based data sharing is one alternative for data sharing in general, and it is a part of the company’s data management. Data spaces provide an interface to external data sources and data use, and such an interface should be an integral part of the company’s data strategy and data management practices. In current approaches, this has been given little attention. Specifications provide APIs for external applications and some support for the management of data product offers and contracts. In our opinion, this is not enough. Sharing confidential data must be in full control of the data rights holder, meaning that management of both offers and contracts must be taken seriously. Our model pays attention to this, but there is a lot remaining to work on.

Data space defines a trustworthy collaboration network if the onboarding process is well-defined and correctly evaluates member candidates' trustworthiness. This opens a trade-off between the ease of being part of the data space and the trustworthiness of the collaboration network. Both extremes and solutions in between will likely be useful in some data space deployments.

**Fig. 20:**
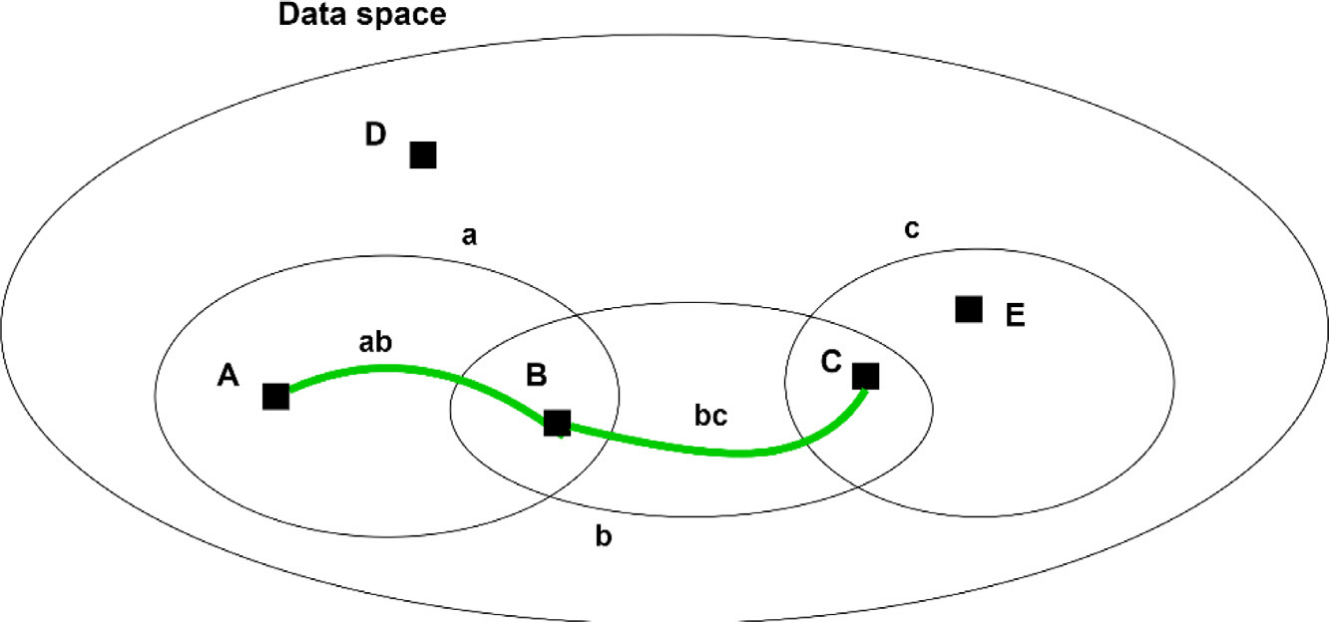
Venn diagram of creating collaboration networks using visibility criteria in data product offers. Member A, B, and C's data product has visibility for sets a, b, and c, respectively. The data-sharing contracts are ab and bc, and they define a potential collaboration network of members (A, B, C, and E) belonging to a union of a, b, and c.

Another more dynamic and versatile option is to use the visibility of data offers to create business collaboration networks. In Fig. 20 we have three members *A, B*, and *C* with data product offers described in sets *a, b*, and *c,* respectively. This means that members in these sets can be aware of these offers and propose data sharing contracts related to them. So, the union of sets *a, b*, and *c* creates a possible collaboration network, but none of the members *A, B, C,* and *E* know all the offers. The actual collaboration takes place only with members that share data. In the example, two contracts, ab and bc, are visible, and members A, B, C are part of the collaboration network. Our model includes this capability that can support defining confidential or secret value creation networks inside the potential network of the whole data space. Still, more research is needed in maximising the potential of the concept even at the logical level.

As described in Fig. 1 data space developments may lead to multiple different technologies and implementation alternatives. It is also likely that solutions with conceptual differences will be called data spaces even when the links to the basic characteristics are weak. The focus chosen in this paper is the trustworthiness of data sharing instead of providing a huge pool of data for use. We believe that secure and sovereignty-preserving data sharing will eventually create a huge pool of data for possible uses. This focus also separates the data spaces from platforms and emphasises the collaboration network views. When the data space services mature, they may merge into the Internet, and we could call a data space a step towards a trustworthy Internet.

## 7 Conclusions

The paper presents a use case and logical architecture models of the data space concept. It looks at the sharing of confidential and sensitive data from the user or data rights holder's perspective and focuses on features that make data sovereignty and trustworthiness a reality. Data sovereignty requires that the data rights holder, or the data provider, fully controls its data, and the data is shared through a secure channel to the receiver, the data consumer.

The logical architecture model divides a data space system into three main components: common rules, creation of trustworthiness, and contract-based data sharing. Common rules are needed to ensure a level playing field for all the stakeholders. Trustworthiness is tackled by knowing the users and their real identities, knowing the components and services involved are what they claim to be, using secure and verifiable communication, and binding the operations to legal frameworks by legal agreements. Contract-based data sharing moves the focus from access and use of data to agreeing and executing contract conditions. All actions that happen in data sharing, happen only when agreed legally binding contract conditions are met.

The main result of this work is the implementation of an independent, simple, and explainable model of data space’s functionality. It highlights the main reasons that data spaces are for and provides a good basis for learning and understanding data spaces. It gives functional requirements to data spaces without biases to any implementations or implementation style and this gives more freedom to implement data spaces that users need. It considers the data space a data sharing system that includes parts that need to be integrated into users’ data management system instead of describing only external services.

The work aligns with current data space approaches. The components of the logical architecture can be mapped easily with concepts, functional model services, and system model components. At the same time, it opens views to see data spaces differently using collaboration networks and contract-based sharing concepts, and eventually, this has the potential to lead to a trustworthy Internet.

## CRediT Author Statement

**Juha-Pekka Soininen**: Conceptualization, Methodology, Writing – original draft preparation, Writing - Review & Editing. **Gabriella Laatikainen**: Conceptualization, Validation, Writing – original draft preparation, Writing - Review & Editing.

## Data Availability

DSSCDSSC website (Reference data).
